# Crystal Structures of PRK1 in Complex with the Clinical Compounds Lestaurtinib and Tofacitinib Reveal Ligand Induced Conformational Changes

**DOI:** 10.1371/journal.pone.0103638

**Published:** 2014-08-11

**Authors:** Philip Chamberlain, Silvia Delker, Barbra Pagarigan, Afshin Mahmoudi, Pilgrim Jackson, Mahan Abbasian, Jeff Muir, Neil Raheja, Brian Cathers

**Affiliations:** 1 Celgene Corporation, San Diego, California, United States of America; 2 Department of Biochemistry and Structural Biology, Celgene Corporation, San Diego, California, United States of America; Monash University, Australia

## Abstract

Protein kinase C related kinase 1 (PRK1) is a component of Rho-GTPase, androgen receptor, histone demethylase and histone deacetylase signaling pathways implicated in prostate and ovarian cancer. Herein we describe the crystal structure of PRK1 in apo form, and also in complex with a panel of literature inhibitors including the clinical candidates lestaurtinib and tofacitinib, as well as the staurosporine analog Ro-31-8220. PRK1 is a member of the AGC-kinase class, and as such exhibits the characteristic regulatory sequence at the C-terminus of the catalytic domain – the ‘C-tail’. The C-tail fully encircles the catalytic domain placing a phenylalanine in the ATP-binding site. Our inhibitor structures include examples of molecules which both interact with, and displace the C-tail from the active site. This information may assist in the design of inhibitors targeting both PRK and other members of the AGC kinase family.

## Introduction

Protein Kinase C-Related Kinase 1 is a protein kinase with roles in Rho- [1]–[3] and androgen receptor mediated signaling [4]. PRK1 and PRK2 have been shown to phosphorylate HDAC5, HDAC7 and HDAC9 with the effect of inhibiting nuclear localization [5]. A further role for PRK1 in the development of germinal centers downstream of the B-cell receptor has also been reported [6]. Clinically, PRK1 has been shown to be overexpressed in ovarian serous carcinoma [7]. Furthermore, PRK1 expression levels correlate with Gleason scores in prostate cancer, and knockdown of PRK1 is anti-proliferative in LNCaP cells [8]. PRK1 is able to shuttle between the cytoplasm and the nucleus and is modulated by RhoA, cardiolipin, arachidonic acid, proteolysis [9], phosphorylation, phosphoinositides [10], and various cell stresses [11]. PRK1 also has roles in the epigenetic regulation of transcription: PRK1 phosphorylates histone H3 at Thr11 [8]. In this way, PRK1 was posited to act as a gatekeeper for androgen dependent transcription by enhancing recruitment of the demethylase JMJD2C, although there are contradictory reports that histone H3 phosphorylation actually inhibits JMJD2C recruitment [12].

The discovery of potent and selective PRK1 inhibitors would provide tools with which to interrogate PRK1 biology, and may pave the way for a clinical PRK1 modulator. Several small molecule inhibitors have been identified as PRK1 inhibitors: The bisindole maleimide compound Ro-31-8220 is a staurosporine analogue which has been shown to have activity against PRK1 [13]. Another staurosporine analogue, lestaurtinib (also known as CEP701), was identified as an inhibitor of PRK1 in a screening effort focusing on clinical candidates [14]. Lestaurtinib inhibits several other protein kinases including FLT3 and JAK2 [15], [16]. As such, lestaurtinib has been evaluated in clinical studies in myelofibrosis and AML [17], [18]. Tofacitinib, a JAK3 inhibitor approved for clinical use in rheumatoid arthritis [19], [20], is particularly interesting as it possesses considerable specificity across the kinome: In a 317 kinase panel, tofacitinib only inhibits 3 kinases with an IC_50_ <500 nM: JAK3, JAK2 and PRK1 [21]. Further small molecule PRK1 inhibitors have been reported as a result of virtual screening using a PRK1 homology model [22].

PRK1 is a member of the AGC kinase family, which also includes PKA, PKC, RSK, SGK, GRK and PKB (AKT). A characteristic feature of the AGC kinases is a C-terminal regulatory region (C-tail) [23]. The C-tail is involved in the regulation of enzyme activity, and can act in the recruitment of binding partners including PDK1 [24], [25]. The C-tail can insert a conserved phenylalanine residue into the ATP-binding site where many kinases exhibit an open solvent channel. This phenylalanine containing region has been described as the active-site tether, and is able to interact with bound nucleotide/inhibitors. In this work we present the crystal structure of PRK1 in the apo state as well as in complex with the staurosporine analogs lestaurtinib and Ro-31-8220, as well as the highly selective inhibitor tofacitinib. We hope this information may accelerate the design of highly selective inhibitors targeting both PRK and other members of the AGC kinase family.

## Materials and Methods

### Expression and purification of PRK1

WT or Phe910Ala human PRK1 residues 611–942 were cloned into PFASTBAC-LIC by ligation independent cloning to generate a transcript incorporating a thrombin cleavable N-terminal His-tag. Bacmids were generated using DH10Bac (Invitrogen). SF9 cells were transfected using Cellfectin (Invitrogen), and viruses were amplified in 3 passages using an automated system (Qiagen). PRK1 was expressed in High5 cells (Invitrogen). Cells were seeded at 2×10 E6/mL into SF921media (Expression Systems) in a 10L wavebag. Cells were shaken at 27°C and harvested 48 hours post-infection. PRK1 was purified by nickel affinity, anion exchange and size exclusion chromatography. Briefly, SF9 cells were resuspended in 25 mM Hepes pH 7.5, 500 mM NaCl, 10 mM Imidazole, 5 mM TCEP, 10% glycerol, 0.1% Brij, EDTA-free protease inhibitor cocktail (San Diego Bioscience) and lysed using a microfluidizer. The lysate was centrifuged at 100,000 g for 1 hr and then applied to a Histrap FF column (GE Healthcare). Protein was eluted with 25 mM Hepes pH 7.5, 500 mM NaCl, 750 mM Imidazole, 5 mM TCEP, 10% glycerol and 0.1% Brij. The fractions containing PRK1 were dialyzed over night in 25 mM Tris-HCl pH 8.0, 5 mM NaCl, and 5 mM DTT in the presence of Thrombin. The cleaved PRK1 was loaded back on Ni-NTA to remove any uncleaved protein then immediately loaded onto a MonoQ anion exchange column (GE Healthcare). The column was washed with 25 mM Tris-HCl pH 8.0, and 5 mM DTT and eluted with a linear gradient from 5 to 1000 mM NaCl. The protein was then loaded on a Superdex 75 size exclusion chromatography column (GE Healthcare) and subsequently concentrated to 10 mg/ml in 25 mM Tris-HCl pH 8.5, 50 NaCl, and 5 mM DTT. MS analysis confirmed that PRK1 was phosphorylated at 2 sites within the expressed region.

### Crystallization of PRK1

PRK1 was crystallized by sitting drop vapor diffusion. Briefly, protein mixed 1∶1 with, and subsequently equilibrated against a reservoir solution of 100 mM Tris pH 8.5, 150–225 mM Ammonium acetate, 23–28% PEG 3350. Lestaurtinib and Ro-31-8220 were obtained from Tocris Bioscience. Tofacitinib was obtained from Selleckchem. Ligand structures were obtained by soaking pre-grown crystals: compounds in DMSO were added directly to the crystal drops 1–24 hours before harvesting. We noted that ligands which displace the C-tail region required substantially longer soaking times, typically around 2 days. Crystals were cryoprotected by addition of 20% PEG200 and frozen under liquid nitrogen.

### Data collection and structure determination

**Figure 1:**
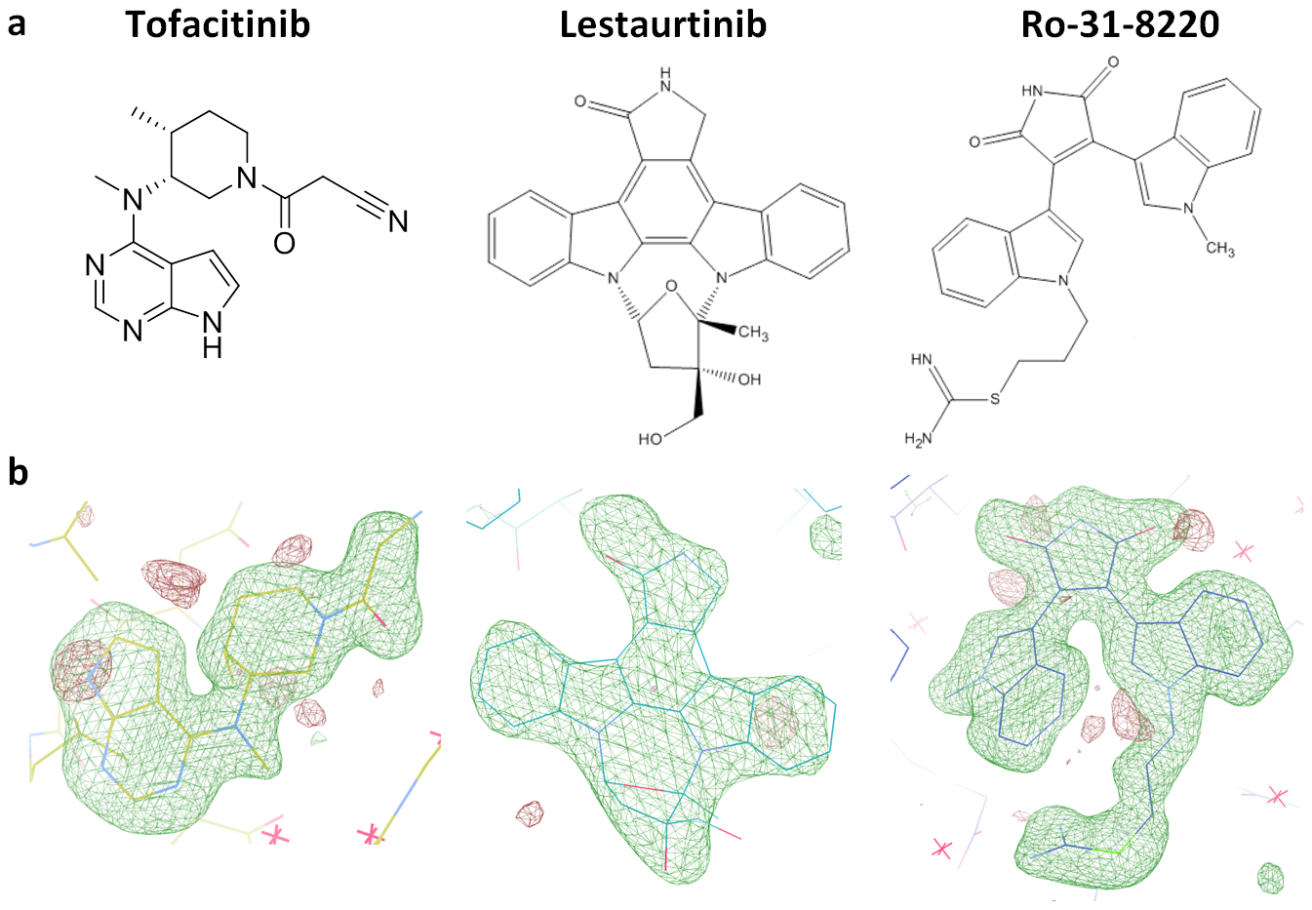
Chemical structures and electron density for PRK1 inhibitors discussed in this paper. 1a, Chemical structures for PRK1 inhibitors. 1b, Difference electron density calculated at 3.5σ following REFMAC5 refinement in the absence of ligand.

Data was collected from single crystals cooled to 100K at the synchrotron beamlines listed in Table 1. Data were integrated and scaled using HKL2000 [26]. The structure of PRK1 was solved by molecular replacement using PHASER [27] using PKCθ as a search model (PDB code 1XJD) [28]. The initial molecular replacement solution was followed by iterative rounds of model building using COOT [29], followed by restrained refinement using REFMAC5 [30]. Final refinement statistics are shown in Table 1, electron density for ligands is shown in Figure 1b. The coordinates and refined data have been deposited in the Protein Data Bank with the accession codes: 4OTD, 4OTG, 4OTH, and 4OTI.

**Table 1:** Crystallographic Statistics for PRK1 datasets.

Ligand	Apo	Tofacitinib	Lestaurtinib	Ro-31-8220
PDB code	4OTD	4OTI	4OTG	4OTH
Data collection site	ALS 5.0.1	ALS 5.0.2	APS(LS-CAT, 21ID-G)	ALS 5.0.3
Wavelength (Å)	1.0	1.0	1.0	1.0
Resolution Range (Å)	50-2.0 (2.03-2.00)*	50-1.93 (1.96-1.93)	50-2.53 (2.62-2.53)	50-1.8 (1.8-1.85)
Spacegroup	*P*2_1_2_1_2_1_	*P*2_1_2_1_2_1_	*P*2_1_2_1_2_1_	*P*2_1_2_1_2_1_
Cell dimensions (Å)	56.2 72.6 94.5	56.3 72.5 94.5	56.5 73.3 94.5	56.1 72.2 94.9
Angles (deg)	90 90 90	90 90 90	90 90 90	90 90 90
No. of observations	89513	88098	71314	112341
No. of unique observations	26396	28110	13264	33917
Completeness (%)	98.2 (88.0)*	94.4 (78.9)	98.1 (99.9)	92.8 (84.1)
*I/σI*	27.0 (4.4)	34.3 (3.6)	13.19 (2.77)	34.0 (2.2)
R_merge (%)_	3.8 (15.6)	5.2 (32.8)	11.9 (65.7)	4.3 (29.5)
**Refinement statistics**				
R_work_/R_free_	17.5/22.1 (19.7/25.4)	18.5/22.6 (21.8/27.7)	20.9/26.4 (27.3/38.3)	20.8/26.6 (28.5/31.1)
RMSD for bond length (Å)	0.013	0.012	0.012	0.010
RMSD for bond angles (deg)	1.564	1.595	1.720	1.281

*Figures in parentheses are for the outer resolution shell.

### Biochemical assay

PRK1 activity was tested in an IMAP-TR FRET format, which detects the phosphorylation of a fluorescently labeled peptide using a phosphate binding probe. PRK1 611–942 in Assay Buffer (50 mM HEPES pH 7.6, 1 mM DTT, 10 mM MgCl2, 0.01% Triton X-100, 0.01% BSA and 0.1 mM EDTA) was added to a Costar 3572 384-well white plate to a final concentration of 2.6 nM. 5-carboxyfluoresceine labeled peptide (Molecular Devices #R7169) was added to a final concentration of 1.5 µM. ATP was added to a final concentration of 3.5 µM, which is our experimentally determined Km for ATP for PRK1, and the mixture was incubated at room temperature for 1 hour. 30 µl/well of Detection Binding Solution (Molecular Devices) was added and incubated overnight at room temperature. The assay plates were read on a PerkinElmer EnVision microplate reader, with the excitation wavelength at 320 nm and emission at 520 nm.

## Results

### Structure of apo PRK1

**Figure 2:**
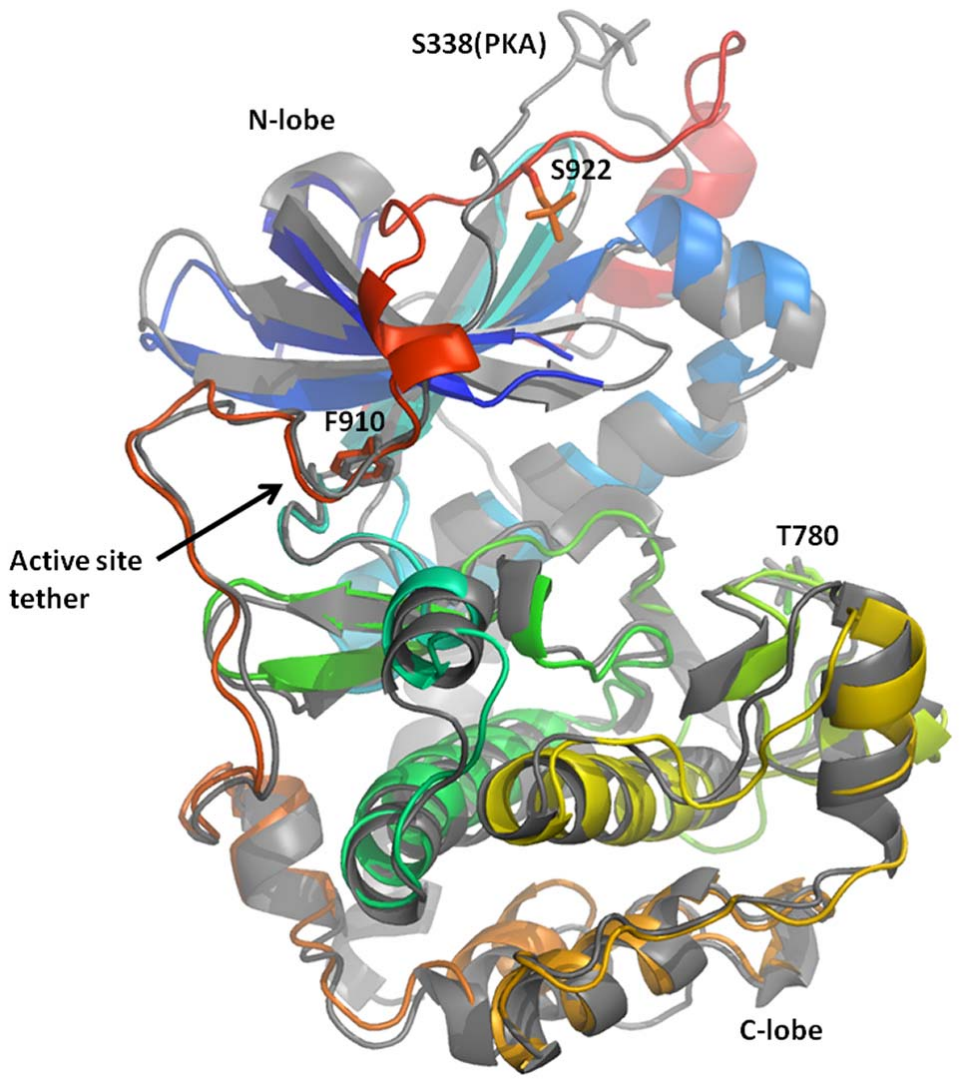
Comparison of PRK1 to PKA. The structure of PRK1 is shown colored from blue to red (N- to C- terminus); PKA (1BKX) is shown in grey. Sites of phosphorylation (PRK1 residues S922 and T780, PKA residue S338) are shown as sticks, as is active site tether residue F910 (PRK1). The structures show good alignment, with a divergence in the conformation of the C-tail as it passes over the N-lobe.

The structure of apo PRK1 has been solved to 2 Å resolution (Table 1). In the apo structure, the entire C-terminal region of the gene containing the catalytic domain and C-tail is fully defined in the electron density. Two phosphorylation sites were determined to be present by mass spectral (MS) analysis (Data not shown), and both of these are clearly visible in the structure, at residues Thr780 in the activation loop and Ser922 in the Turn-motif (Figure 2). Structural alignment with PKA yields a 1.8 Å RMSD over 270 aligned residues (PKA coordinates 1BKX, Figure 2).

The C-tail region begins at the C-terminus of the catalytic domain, which is found at the base of the C-Lobe as shown in Figure 2. From here, the C-tail follows a route around the surface of the C-lobe, and passes adjacent to the kinase ‘hinge’ before continuing to encircle the N-lobe. The phosphorylation site at Ser922 is buried in a charged pocket formed on the N-lobe from residues Arg629, Lys634 and Lys653. The site of the phosphorylation is structurally homologous with PKCθ, AKT, S6K, RSK, MSK, PKC iota but different from that found in PKA (Figure 2) [31], [32].

Crystal structures of PKA have also exhibited a fully ordered C-Tail [33]. Alignment of PKA with PRK1 shows that there is close alignment between the C-tails up to PRK1 residue 911, which includes the conserved ‘active site tether’ residue Phe910. The conformation of the respective C-tails around the N-Lobe diverges up to >9 Å across the surface of the N-lobe, with structural conservation restored at the hydrophobic motif (Figure 2). Many AGC kinases require phosphorylation in the hydrophobic motif prior to recruitment of PDK1 as part of the activation sequence [25], however, PRK1 has an aspartate residue (Asp942) which may mimic the phosphorylation thereby bypassing this regulatory step.

The PRK1 C-Tail residue Phe910 projects into the enzyme active site adjacent to the kinase hinge. In this position, the Phe910 sidechain is stacked between the sidechain of Leu627 and Gly707, with further Van der Waals interactions from the side of the phenylalanine ring to the kinase ‘hinge’ residue Tyr703. Kinase active sites are typically open to solvent at this position, and ATP competitive kinase inhibitors commonly bind the hinge region proximal to the sidechain of Phe910.

### Structures of the staurosporine analogs lestaurtinib and Ro-31-8220

**Figure 3:**
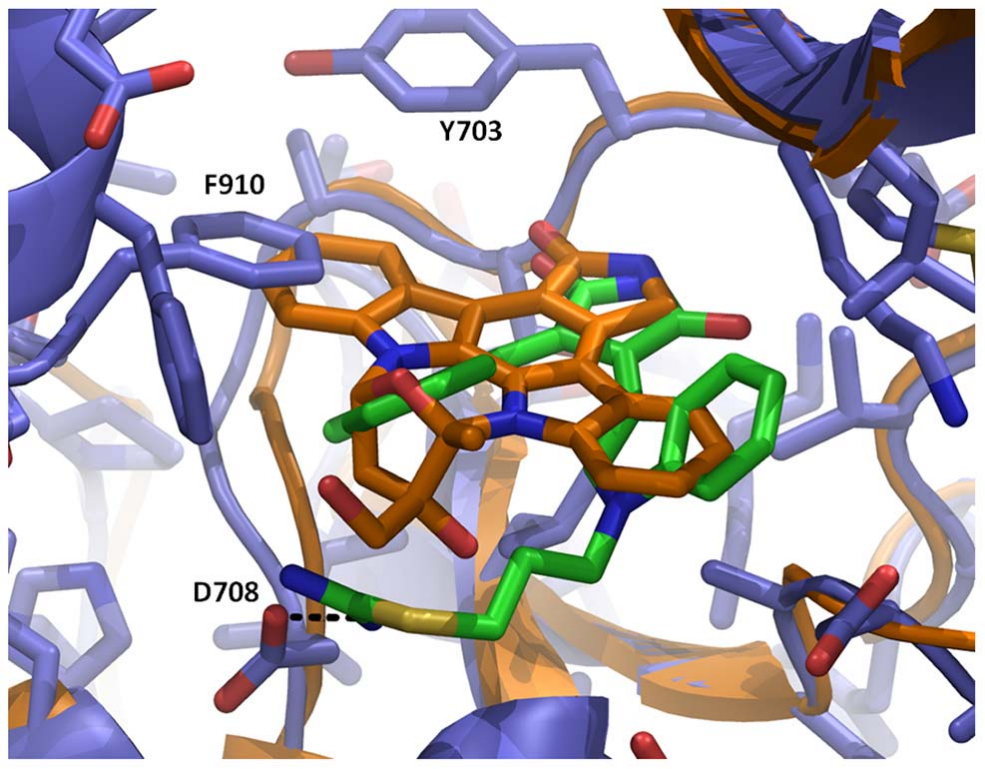
Comparison of the binding modes of Ro-31-8220 and lestaurtinib showing that lestaurtinib and F910 would undergo a steric clash without protein motion. PRK1:Ro-31-8220 is shown with the protein in blue, and the inhibitor in green. PRK1:Lestaurtinib is shown colored orange. Side chains are shown only for the PRK1:Ro-31-8220 structure.

**Figure 4:**
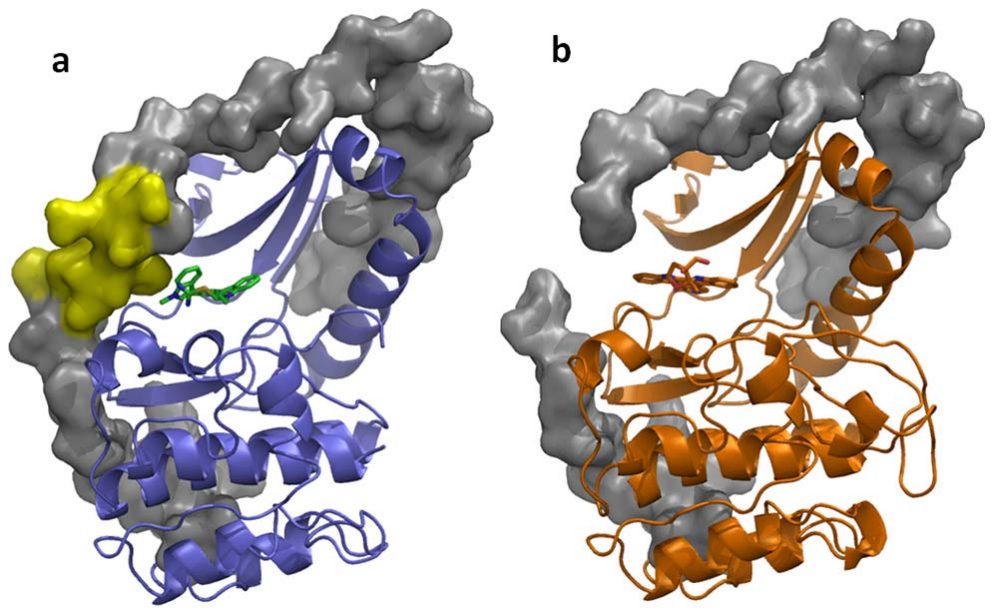
Disordering of the PRK1 C-tail in the lestaurtinib crystal structure. (a) PRK1:Ro-31-8220 is shown with the protein in blue, with the inhibitor in green. The C-tail is shown as a surface in gray; the region disordered in the lestaurtinib structure is shown in yellow on the Ro-31-8220 structure. (b) PRK1:Lestaurtinib is shown colored orange with the C-tail shown as a surface in grey, with a gap visible due to disorder of the C-tail proximal to the inhibitor.

Staurosporine, lestaurtinib and Ro-31-8220 inhibit PRK1 with IC50s of 2 nM, 27 nM and 9 nM, respectively (Table 2). The structures of the two staurosporine analogues in complex with PRK1 are consistent with the known literature binding modes for this ligand class, with two hydrogen bonds between the ligand and the kinase hinge. A further hydrogen bond is made by Ro-31-8220 to Asp708 in the ribose pocket (Figure 3). In the structure with Ro-31-8220, the 2 indole rings are able to rotate to achieve a compact structure able to fit into the PRK1 binding site as it occurs in the apo structure. However, the indole ring in lestaurtinib is constrained to a more planar structure (Figure 1) which cannot be accomodated in the PRK1 binding site without conformational changes. As shown in the structural superposition between lestaurtinib and Ro-31-8220 (Figure 3), lestaurtinib clashes with the C-tail residue Phe910 where Ro-31-8220 is able to rotate the indole rings into a conformation in which there is no steric clash. In the bound conformation, Ro-31-8220 is able to form hydrophobic interactions with Phe910. In contrast, a substantial disordering of the C-tail occurs upon binding of lestaurtinib, with 12–14 residues from the active site tether region of the C-tail no longer visible in the electron density (Figure 4). Despite the fact that lestaurtinib appears to displace a substantial region of the protein from the ATP-binding site, lestaurtinib is only ∼3-fold less potent than Ro-31-8220 (Table 2). To determine the effect of Phe910 displacement on ligand potency, we mutated Phe910 to an alanine. Phe910Ala mutant PRK1 exhibited no significant difference in activity to WT and exhibited a similar Km for ATP (data not shown). As shown in Table 2, when assayed under the same conditions there are no significant differences in IC_50_ values between the mutant and the WT protein.

**Table 2:** IC50 values (nM) for PRK1.

Compound	WT	F910A
Lestaurtinib	26 (±0.7)	29 (±0.8)
Ro-31 8220	9 (±3)	9 (±2)
Staurosporine	2.0 (0.07)	3 (±0.1)
Tofacitinib	43 (±4)	58 (±10)

Values are the average of more than 3 replicate experiments, standard deviations are shown in parentheses.

### Structure of PRK1 in complex with tofacitinib

**Figure 5:**
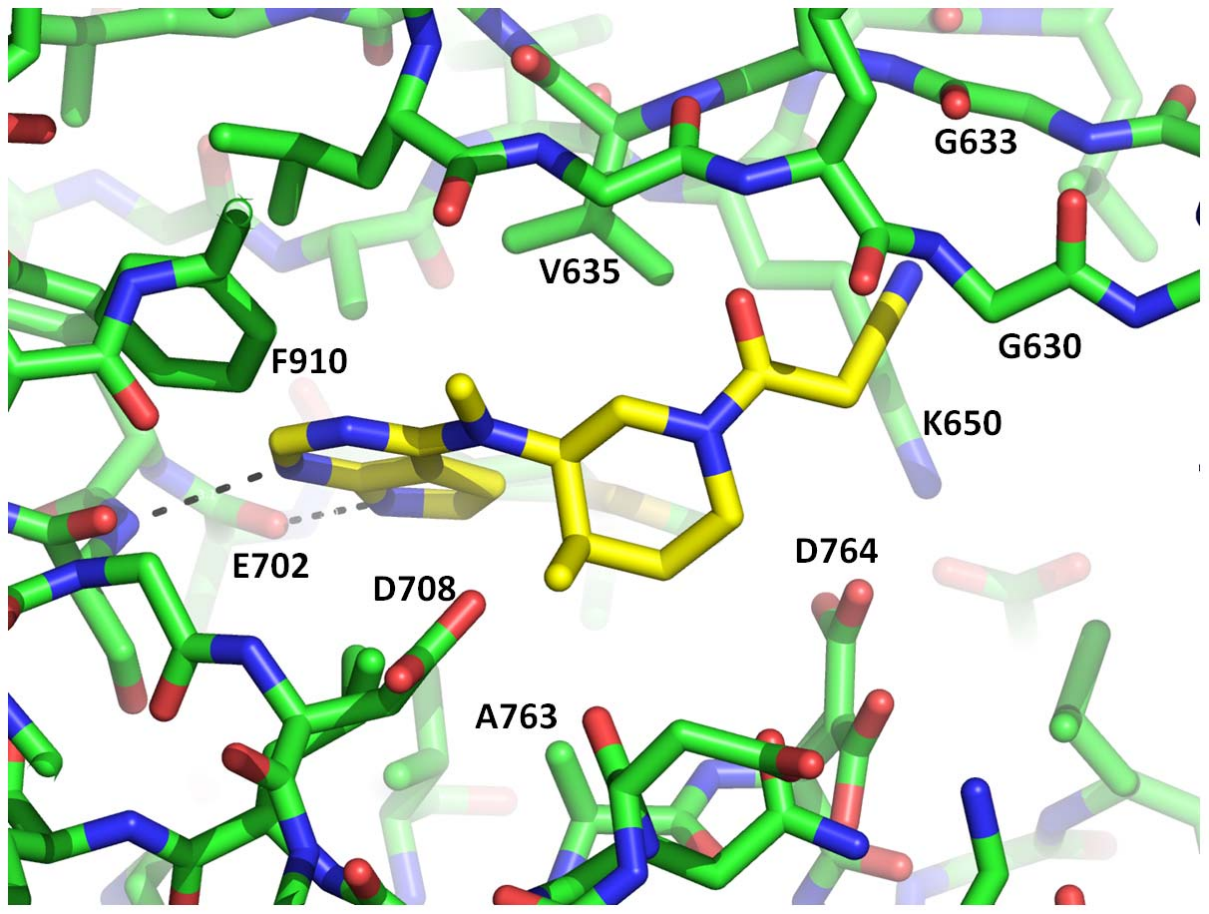
Tofacitinib bound in the active site of PRK1. The protein is shown with carbons represented in green, the ligand with carbons represented in yellow. 2 hydrogen bonds to the kinase hinge are shown as dotted lines.

**Figure 6:**
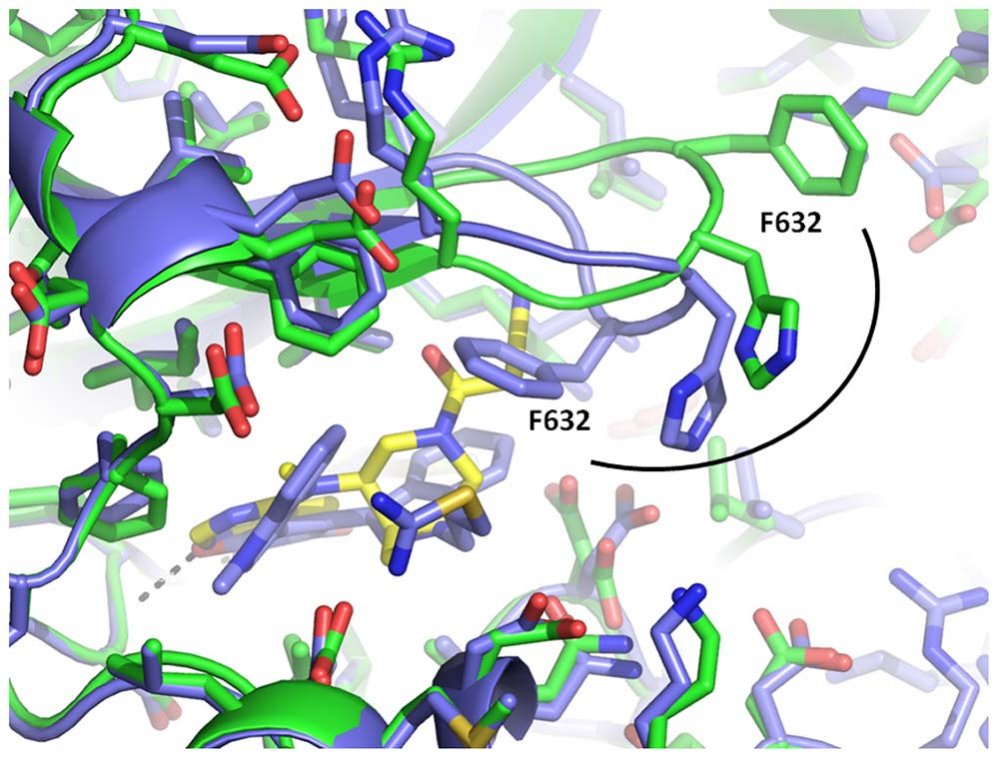
Structural alignment between tofacitinib and Ro-31-8220 showing a large shift in the conformation of G-loop residue F632 between the 2 structures. In the tofacitinib structure the protein is shown with carbons in green, the ligand with carbons in yellow. The Ro-31-8220 structure is shown with carbons in blue.

Tofacitinib binds to PRK1 in a classical type-I binding mode forming two hydrogen bonds between the kinase hinge and the inhibitor pyrrolopyrazine group (Figure 5). Tofacitinib does not displace Phe910 from the active site, and the C-tail is well defined in the structure. Tofacitinib makes substantial van der Waals interactions with the sidechain of Phe910 from the pyrrolopyrazine hinge-binding motif and also from the proximal amino-methyl group. However, tofacitinib does displace another phenylalanine residue from the kinase active site: the ligand nitrile group reaches up to the G-loop and binds in a small groove between the G-loop backbone and the sidechain of the catalytic lysine residue Lys650. In this position the tofacitinib nitrile group would clash with the sidechain of G-loop residue Phe632 in the conformation observed in other structures (Figure 6). Phe632 provides hydrophobic interactions with lestaurtinib, and Ro-31-8220, but as shown in Figure 6, in the tofacitinib structure Phe632 has swung away into solvent ∼12 Å beyond the possibility of ligand interactions.

With Phe632 positioned away from the ATP binding site, the tofacitinib alpha cyano acetamide moiety contacts both beta-strands of the G-loop at residues Gly630 and Gly633 (Figure 5). Adjacent to this moiety, the inhibitor piperidine ring bridges between the G-loop contacts at the ‘top’ of the kinase pocket and the base of the pocket via Van der Waals interactions to Ala763 (4.0 Å). An alanine or glycine residue occurs at the equivalent position in 45% of kinases [34], which means that 55% of kinases possess bulkier residues that would sterically clash with tofacitinib. The simultaneous contact with both the G-loop and the base of the ATP binding pocket via a relatively rigid chemical structure likely underlies some proportion of the kinome selectivity exhibited by tofacitinib. To test this hypothesis, similar structural features could be replicated on other inhibitor scaffolds to attempt to engineer improved selectivity.

## Discussion

In this work we have described the structure of PRK1- a protein kinase overexpressed and mechanistically implicated in prostate and ovarian cancer. Crystal structures of PRK1 in complex with ATP-competitive ligands show that the binding site is capable of adapting to ligands with motions in both the G-loop and C-tail.

Since Phe910 projects into the active site it could influence both substrate and inhibitor binding. However, comparing the IC_50_s between compounds which displace Phe910 and those which do not, there is no discernible difference in potency. The same is true when comparing inhibitor IC_50_s between WT PRK1 and Phe910Ala mutant PRK1. The high degree of sequence conservation at this site indicates an important role for Phe910 in the AGC kinases, but the extent to which members of the class exhibit the Phe910-in conformation *in vivo* remains to be determined. We have observed that the soaking times required to obtain liganded structures are much greater for ligands which displace Phe910: >1 day versus 1 hour. It is therefore possible that the C-tail conformation observed is weakly maintained or transient in nature in solution, or otherwise an artifact of crystallization with little relevance to the protein conformation *in vivo*. It is also possible that intracellular binding partners may stabilize the C-tail conformation in a similar manner to the crystal lattice, resulting in changes in ligand binding in the cellular environment that may not be observed biochemically. Regardless, it would seem prudent to monitor structure-activity relationships of small molecule inhibitors with modifications in the solvent channel proximal to Phe910 in drug discovery programs targeting AGC kinases.

The biology of PRK1 remains to be further studied, and potent and selective small molecule inhibitors may enable and accelerate this work. Here we have presented the structure of tofacitinib, which exhibits a biochemical potency of ∼40 nM and a high level of kinome selectivity. Tofacitinib may therefore present a superior tool for investigating the biological role of PRK1. Furthermore, the ligand structures we have described in complex with PRK1 may provide insights to enable the development of highly selective PRK1 inhibitors.

## References

[1] Amano M, Mukai H, Ono Y, Chihara K, Matsui T, et al (1996) Identification of a putative target for Rho as the serine-threonine kinase protein kinase N. Science 271: 648–650. 8571127 10.1126/science.271.5249.648

[2] Maesaki R, Ihara K, Shimizu T, Kuroda S, Kaibuchi K, et al (1999) The structural basis of Rho effector recognition revealed by the crystal structure of human RhoA complexed with the effector domain of PKN/PRK1. Mol Cell 4: 793–803. 10619026 10.1016/s1097-2765(00)80389-5

[3] Watanabe G, Saito Y, Madaule P, Ishizaki T, Fujisawa K, et al (1996) Protein kinase N (PKN) and PKN-related protein rhophilin as targets of small GTPase Rho. Science 271: 645–648. 8571126 10.1126/science.271.5249.645

[4] Metzger E, Muller JM, Ferrari S, Buettner R, Schule R (2003) A novel inducible transactivation domain in the androgen receptor: implications for PRK in prostate cancer. EMBO J 22: 270–280. 12514133 10.1093/emboj/cdg023 PMC140098

[5] Harrison BC, Huynh K, Lundgaard GL, Helmke SM, Perryman MB, et al (2010) Protein kinase C-related kinase targets nuclear localization signals in a subset of class IIa histone deacetylases. FEBS Lett 584: 1103–1110. 20188095 10.1016/j.febslet.2010.02.057

[6] Yasui T, Sakakibara-Yada K, Nishimura T, Morita K, Tada S, et al (2012) Protein kinase N1, a cell inhibitor of Akt kinase, has a central role in quality control of germinal center formation. Proc Natl Acad Sci U S A 109: 21022–21027. 23223530 10.1073/pnas.1218925110 PMC3529033

[7] Galgano MT, Conaway M, Spencer AM, Paschal BM, Frierson HF Jr (2009) PRK1 distribution in normal tissues and carcinomas: overexpression and activation in ovarian serous carcinoma. Hum Pathol 40: 1434–1440. 19427017 10.1016/j.humpath.2009.02.008 PMC2744839

[8] Metzger E, Yin N, Wissmann M, Kunowska N, Fischer K, et al (2008) Phosphorylation of histone H3 at threonine 11 establishes a novel chromatin mark for transcriptional regulation. Nat Cell Biol 10: 53–60. 18066052 10.1038/ncb1668 PMC2878724

[9] Takahashi M, Mukai H, Toshimori M, Miyamoto M, Ono Y (1998) Proteolytic activation of PKN by caspase-3 or related protease during apoptosis. Proc Natl Acad Sci U S A 95: 11566–11571. 9751706 10.1073/pnas.95.20.11566 PMC21681

[10] Palmer RH, Dekker LV, Woscholski R, Le Good JA, Gigg R, et al (1995) Activation of PRK1 by phosphatidylinositol 4,5-bisphosphate and phosphatidylinositol 3,4,5-trisphosphate. A comparison with protein kinase C isotypes. J Biol Chem 270: 22412–22416. 7673228 10.1074/jbc.270.38.22412

[11] Mukai H, Miyahara M, Sunakawa H, Shibata H, Toshimori M, et al (1996) Translocation of PKN from the cytosol to the nucleus induced by stresses. Proc Natl Acad Sci U S A 93: 10195–10199. 8816775 10.1073/pnas.93.19.10195 PMC38360

[12] Lohse B, Helgstrand C, Kristensen JB, Leurs U, Cloos PA, et al (2013) Posttranslational modifications of the histone 3 tail and their impact on the activity of histone lysine demethylases in vitro. PLoS One 8: e67653. 23844048 10.1371/journal.pone.0067653 PMC3699631

[13] Standaert M, Bandyopadhyay G, Galloway L, Ono Y, Mukai H, et al (1998) Comparative effects of GTPgammaS and insulin on the activation of Rho, phosphatidylinositol 3-kinase, and protein kinase N in rat adipocytes. Relationship to glucose transport. J Biol Chem 273: 7470–7477. 9516446 10.1074/jbc.273.13.7470

[14] Kohler J, Erlenkamp G, Eberlin A, Rumpf T, Slynko I, et al (2012) Lestaurtinib inhibits histone phosphorylation and androgen-dependent gene expression in prostate cancer cells. PLoS One 7: e34973. 22532837 10.1371/journal.pone.0034973 PMC3332061

[15] Hexner EO, Serdikoff C, Jan M, Swider CR, Robinson C, et al (2008) Lestaurtinib (CEP701) is a JAK2 inhibitor that suppresses JAK2/STAT5 signaling and the proliferation of primary erythroid cells from patients with myeloproliferative disorders. Blood 111: 5663–5671. 17984313 10.1182/blood-2007-04-083402 PMC2424161

[16] Levis M, Allebach J, Tse KF, Zheng R, Baldwin BR, et al (2002) A FLT3-targeted tyrosine kinase inhibitor is cytotoxic to leukemia cells in vitro and in vivo. Blood 99: 3885–3891. 12010785 10.1182/blood.v99.11.3885

[17] Knapper S, Burnett AK, Littlewood T, Kell WJ, Agrawal S, et al (2006) A phase 2 trial of the FLT3 inhibitor lestaurtinib (CEP701) as first-line treatment for older patients with acute myeloid leukemia not considered fit for intensive chemotherapy. Blood 108: 3262–3270. 16857985 10.1182/blood-2006-04-015560

[18] Santos FP, Kantarjian HM, Jain N, Manshouri T, Thomas DA, et al (2010) Phase 2 study of CEP-701, an orally available JAK2 inhibitor, in patients with primary or post-polycythemia vera/essential thrombocythemia myelofibrosis. Blood 115: 1131–1136. 20008298 10.1182/blood-2009-10-246363 PMC4081385

[19] Chrencik JE, Patny A, Leung IK, Korniski B, Emmons TL, et al (2010) Structural and thermodynamic characterization of the TYK2 and JAK3 kinase domains in complex with CP-690550 and CMP-6. J Mol Biol 400: 413–433. 20478313 10.1016/j.jmb.2010.05.020

[20] Flanagan ME, Blumenkopf TA, Brissette WH, Brown MF, Casavant JM, et al (2010) Discovery of CP-690,550: a potent and selective Janus kinase (JAK) inhibitor for the treatment of autoimmune diseases and organ transplant rejection. J Med Chem 53: 8468–8484. 21105711 10.1021/jm1004286

[21] Karaman MW, Herrgard S, Treiber DK, Gallant P, Atteridge CE, et al (2008) A quantitative analysis of kinase inhibitor selectivity. Nat Biotechnol 26: 127–132. 18183025 10.1038/nbt1358

[22] Slynko I, Scharfe M, Rumpf T, Eib J, Metzger E, et al (2014) Virtual screening of PRK1 inhibitors: ensemble docking, rescoring using binding free energy calculation and QSAR model development. J Chem Inf Model 54: 138–150. 24377786 10.1021/ci400628q

[23] Kannan N, Haste N, Taylor SS, Neuwald AF (2007) The hallmark of AGC kinase functional divergence is its C-terminal tail, a cis-acting regulatory module. Proc Natl Acad Sci U S A 104: 1272–1277. 17227859 10.1073/pnas.0610251104 PMC1783090

[24] Balendran A, Biondi RM, Cheung PC, Casamayor A, Deak M, et al (2000) A 3-phosphoinositide-dependent protein kinase-1 (PDK1) docking site is required for the phosphorylation of protein kinase Czeta (PKCzeta) and PKC-related kinase 2 by PDK1. J Biol Chem 275: 20806–20813. 10764742 10.1074/jbc.M000421200

[25] Biondi RM, Komander D, Thomas CC, Lizcano JM, Deak M, et al (2002) High resolution crystal structure of the human PDK1 catalytic domain defines the regulatory phosphopeptide docking site. EMBO J 21: 4219–4228. 12169624 10.1093/emboj/cdf437 PMC126174

[26] Otwinowski Z, Minor W (1997) Processing of X-ray Diffraction Data Collected in Oscillation Mode. Methods in Enzymology 276: 307–326. 10.1016/S0076-6879(97)76066-X 27754618

[27] McCoy AJ, Grosse-Kunstleve RW, Adams PD, Winn MD, Storoni LC, et al (2007) Phaser crystallographic software. J Appl Crystallogr 40: 658–674. 19461840 10.1107/S0021889807021206 PMC2483472

[28] Xu ZB, Chaudhary D, Olland S, Wolfrom S, Czerwinski R, et al (2004) Catalytic domain crystal structure of protein kinase C-theta (PKCtheta). J Biol Chem 279: 50401–50409. 15364937 10.1074/jbc.M409216200

[29] Emsley P, Cowtan K (2004) Coot: model-building tools for molecular graphics. Acta Crystallogr D Biol Crystallogr 60: 2126–2132. 15572765 10.1107/S0907444904019158

[30] Murshudov GN, Skubak P, Lebedev AA, Pannu NS, Steiner RA, et al (2011) REFMAC5 for the refinement of macromolecular crystal structures. Acta Crystallogr D Biol Crystallogr 67: 355–367. 21460454 10.1107/S0907444911001314 PMC3069751

[31] Hauge C, Antal TL, Hirschberg D, Doehn U, Thorup K, et al (2007) Mechanism for activation of the growth factor-activated AGC kinases by turn motif phosphorylation. EMBO J 26: 2251–2261. 17446865 10.1038/sj.emboj.7601682 PMC1864980

[32] Messerschmidt A, Macieira S, Velarde M, Badeker M, Benda C, et al (2005) Crystal structure of the catalytic domain of human atypical protein kinase C-iota reveals interaction mode of phosphorylation site in turn motif. J Mol Biol 352: 918–931. 16125198 10.1016/j.jmb.2005.07.060

[33] Narayana N, Cox S, Nguyen-huu X, Ten Eyck LF, Taylor SS (1997) A binary complex of the catalytic subunit of cAMP-dependent protein kinase and adenosine further defines conformational flexibility. Structure 5: 921–935. 9261084 10.1016/s0969-2126(97)00246-3

[34] Vulpetti A, Bosotti R (2004) Sequence and structural analysis of kinase ATP pocket residues. Farmaco 59: 759–765. 15474052 10.1016/j.farmac.2004.05.010

